# A new species of *Oomyzus* Rondani (Hymenoptera, Eulophidae) and first record of *O. gallerucae* (Fonscolombe) from China, with a key to Chinese species

**DOI:** 10.3897/zookeys.950.48795

**Published:** 2020-07-20

**Authors:** Wen-Jian Li, Cheng-De Li

**Affiliations:** 1 School of Forestry, Northeast Forestry University, Harbin, 150040, China Northeast Forestry University Harbin China

**Keywords:** Chalcidoidea, new species, taxonomy, Tetrastichinae

## Abstract

*Oomyzus flavotibialis***sp. nov.** is described from Liaoning and Shandong provinces, China. *Oomyzus gallerucae* (Fonscolombe) is reported for the first time from China. A key to Chinese species of *Oomyzus* is provided.

## Introduction

The genus *Oomyzus* currently contains 26 valid species worldwide (Noyes 2019), with only four species known from China: *O. scaposus* (Thomson), *O. sokolowskii* (Kurdjumov), *O. sinensis*﻿ Sheng & Zhu, and *O. hubeiensis*﻿ Sheng & Zhu (Li 1984; Sheng and Zhu 1998; Wang et al. 1998). Most species of the genus are parasitoids of Coleoptera, Neuroptera, Diptera, and Lepidoptera. They attack larvae or pupae, and even eggs of their hosts, and several species were widely used in classical biological control (Yaseen 1978; Alam 1982; LaSalle 1994; Liu et al. 2000).

*Oomyzus* can be recognized by the following combination of characteristics (Graham 1991; LaSalle 1994): female antenna usually with funicle segments not or slightly longer than wide (rarely funicle segments relatively longer); mesoscutum with 2–5 adnotaular setae on each side; submedian grooves usually weak or absent, rarely strong; propodeum with or without paraspiracular carinae (but not as in *Tetrastichus*); meso- and metabasitarsus often shorter than second tarsomere; forewing with one rarely two, dorsal seta on submarginal vein; metasoma subcircular to ovate, from shorter than mesosoma to about as long as head and mesosoma combined.

In the present paper, a new species of *Oomyzus* from China is described, *O. gallerucae* is newly reported from China, and a key to Chinese species is provided.

## Materials and methods

Specimens were collected by sweep net and most were dissected and mounted dorsally in Canada balsam on steps following the method of Noyes (1982). Photographs were taken with a digital CCD camera attached to an Olympus BX51 compound microscope, and most measurements were made from slide-mounted specimens using an eye-piece reticle with an Olympus CX21 microscope. Terminology follows the Hymenoptera Anatomy Consortium (2019), and the following abbreviations are used: F1–4 (flagellomeres 1–4), POL (minimum distance between lateral ocelli), OOL (minimum distance between lateral ocellus and eye margin), OD (longest diameter of a lateral ocellus), MV (marginal vein), STV (stigmal vein), SMV (submarginal vein). All the specimens listed below are deposited in Northeast Forestry University, Harbin (**NEFU**), China.

### Key to species of *Oomyzus* Rondani from China. Females.

**Table:** 

1	Mid lobe of mesoscutum without median line (Fig. 3)	**2**
–	Mid lobe of mesoscutum with median line present although sometimes weak	**3**
2	MV 2.1–2.6× as long as STV (Fig. 4); propodeum with median carina poorly defined; reticulation absent (Fig. 3)	***O. flavotibialis* sp. nov.**
–	MV 3.0–3.7× as long as STV; propodeum with median carina sharply defined; reticulation present	***O. scaposus* (Thomson)**
3	Callus with 5–8 setae; F1 as long as pedicel	**4**
–	Callus with 2 setae; F1 shorter than pedicel	**5**
4	Propodeum with median carina (see Sheng and Zhu 1998: fig. 7)	***O. sinensis* Sheng & Zhu**
–	Propodeum without median carina (see Sheng and Zhu 1998: fig. 4)	***O. hubeiensis* Sheng & Zhu**
5	Clava 2.0–2.25× as long as broad; meso- and metabasitarsus hardly shorter than corresponding second tarsomeres	***O. sokolowskii* (Kurdjumov)**
–	Clava 2.7× as long as broad (Fig. 10); meso- and metabasitarsus distinctly shorter than corresponding second tarsomeres (Fig. 14)	***O. gallerucae* (Fonscolombe)**


#### Oomyzus flavotibialis sp. nov.

Taxon classificationAnimaliaHymenopteraEulophidae

90BDE698-A2BD-5B0C-8DD1-5C5F05D59974

http://zoobank.org/A6DC6119-AF2F-4A2E-AF3D-35BBFA2EFC91

Figures 1–6 , 7–8

##### Type material.

***Holotype***: female [on slide], China, Liaoning Province, Anshan City, Mount Qian Shan, 23.VI.2015, Hui Geng, Yan Gao, Zhi-Guang Wu, and Si-Zhu Liu, by sweeping. Deposited in NEFU.

***Paratypes***: 8 females, 1 male. China. **Liaoning Province**, same data as holotype, [1 female on card]; Anshan City, Mount Qian Shan, 25.VI.2015, Hui Geng, Yan Gao, Si-Zhu Liu, and Zhi-Guang Wu, sweeping, [2 females on slides]; Anshan City, Mount Qian Shan, 20.IX.2015, Hui Geng, Yan Gao, and Xin-Yu Zhang, sweeping, [2 females on slides]; Fushun City, 18.VI.2012, Hui Geng, Xiang-Xiang Jin, and Jiang Liu, sweeping, [1 female on slide]; **Shandong Province**, Pingdu City, Mount Daze, 18.VII.2014, Hui Geng, Yan Gao, Si-Zhu Liu, and Zhi-Guang Wu, sweeping, [2 females, 1 male on slides]. All deposited in NEFU.

##### Diagnosis.

**Female.** Body black with all tibiae yellow; propodeum with median carina poorly defined, spiracle circular, partly exposed, and separated by about its diameter from metanotum; propodeum reticulation absent; MV 2.1–2.6× as long as STV; SMV with two dorsal setae. *Male.* Antenna with plaque 0.67× as long as scape. Forewing with costal cell 1.4× as long as MV, MV 2.5× as long as STV, SMV with two dorsal setae.

The new species belongs to the *incertus*-group (Graham 1991). Among the species recorded from China, *O. flavotibialis* is similar to *O. scaposus*, but can be separated from the latter by the following combination of characteristics: propodeum with median carina poorly defined, (vs sharply defined), spiracle circular, partly exposed (vs suboval, fully exposed) and separated by about its diameter from metanotum (vs about 0.5× its diameter) ; propodeum reticulation absent (vs present but very fine); MV 2.1–2.6× as long as STV (vs 3.0–3.7×); SMV with two dorsal setae (vs usually with only one); tibiae completely yellow (vs mainly brown to blackish). The new species is also similar to the extralimital species *O. incertus* (see Graham 1991 for description), but can be separated from the latter by characteristics: POL 2.1–2.5× OOL (vs 1.5–1.65×), OOL 1.5–1.8× OD (vs 2.3–2.5×); propodeum medially distinctly longer than dorsellum (vs hardly longer than).

##### Description.

**Female. *Body length*** 1.0–1.2 mm (1.1 mm), black with dark-green metallic reflection. Antenna with radicle dark brown, scape mostly yellow, brown along dorsal edge, pedicel with dorsal half brown, ventral half yellow, flagellum yellowish brown. Metasoma oval, smooth, with weak bronze and bluish tint like mesosoma. Wings hyaline, venation yellowish brown. Legs with coxae dark brown, trochanters yellowish brown; basal 2/3 of pro- and metafemora brown with distal 1/3 yellow, a little more than half of mesofemora yellowish brown basally, remaining distal part yellow; tibiae and basal three tarsomeres yellow, last tarsomeres brown to dark brown.

**Figures 1–6.**
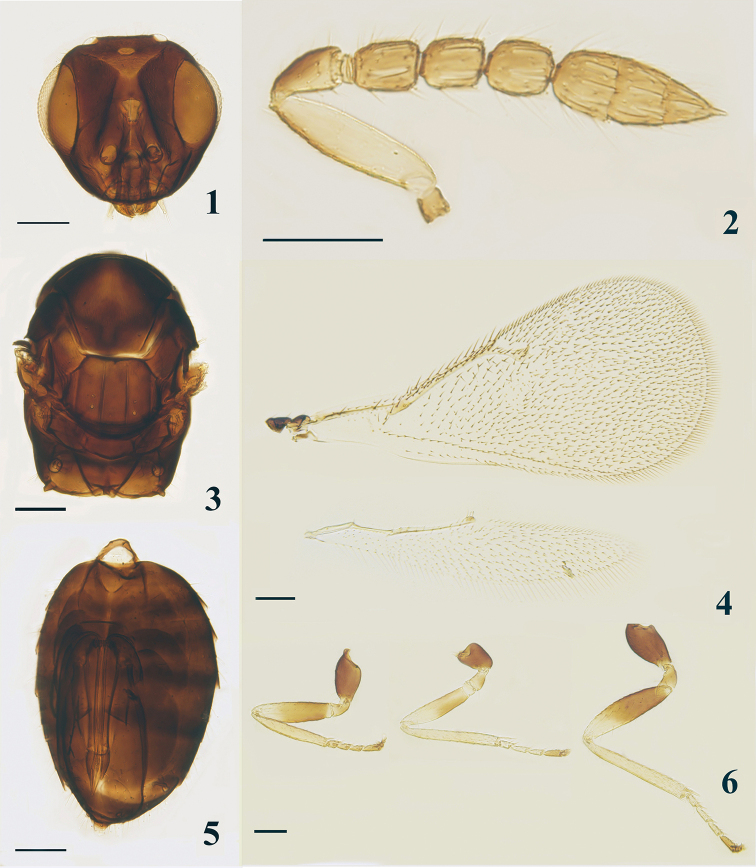
*Oomyzus flavotibialis* sp. nov. holotype, female **1** head, frontal view **2** antenna, lateral view **3** mesosoma, dorsal view **4** fore- and hind wings, dorsal view **5** metasoma, ventral view **6** legs, lateral view. Scale bars: 100 μm.

***Head*** (Fig. 1) in dorsal view, slightly broader than mesosoma with slightly raised reticulation, 3.0–3.4× (3.2×) as broad as long; POL 2.1–2.5× (2.2×) OOL, OOL 1.5–1.8× (1.8×) OD. Malar space 0.53× as long as eye, malar sulcus straight; mouth cavity 1.45× as wide as malar space. Clypeus with anterior margin weakly bidentate. Facial depression moderate, with weak but slightly raised reticulation. Vertex with setae slightly shorter than OD. Torulus with lower edge a little below the ventral edge of eyes. Antenna (Fig. 2) with scape 3.8× as long as broad, shorter than an eye length and not reaching vertex; pedicel longer than F1, 1.7–2.0× (1.9×) as long as broad; three anelli; F1–F3 1.1–1.3× (1.2×), 1.0–1.2× (1.2×), and 1.0–1.2× (1.2×) as long as broad respectively; clava broader than F3, 2.0–2.7× (2.7×) as long as broad, longer than F2 and F3 combined, sensilla moderately numerous, relatively long.

***Mesosoma*** (Fig. 3) 1.3–1.7× (1.4×) as long as broad. Pronotum short, arched. Mid-lobe of mesoscutum 1.2× as broad as long, without median line, with two or three adnotaular setae on each side in one row. Scutellum 1.2× as broad as long; anterior pair of setae in middle, submedian grooves and sublateral grooves distinct, distance between submedian grooves equal to submedian grooves to sublateral grooves. Mesoscutum with extremely fine reticulation, scutellum with similar but finer sculpture. Dorsellum about 2.5× as broad as long. Propodeum medially distinctly longer than dorsellum, smooth and reticulation absent; median carina poorly defined, not distinct; without plicae or traces of plicae at hind margin; spiracle circular, moderate in size and partly exposed, separated from metanotum by about its diameter; callus with four or five setae arranged irregularly. Forewing (Fig. 4) 2.0–2.1× (2.1×) as long as broad, with costal cell 1.1–1.5× (1.5×) as long as MV, MV 2.1–2.6× (2.1×) as long as STV; SMV with two dorsal setae; speculum medium-sized, closed posteriorly. Hind wing (Fig. 4) 4.5–4.8× (4.8×) as long as broad. Legs (Fig. 6) of medium length and thickness, meso- and metabasitarsus distinctly shorter than (0.6–0.7×) corresponding second tarsomeres.

**Metasoma** (Fig. 5) ovate, slightly depressed dorsally, as long as or slightly longer than mesosoma, 1.5–1.7× (1.6×) as long as broad; cercal setae subequal in length. Ovipositor originated from about basal third of gaster, about 0.6× as long as gaster and not exerted at apex, third valvula 0.22× as long as second valvifer.

**Figures 7, 8.**
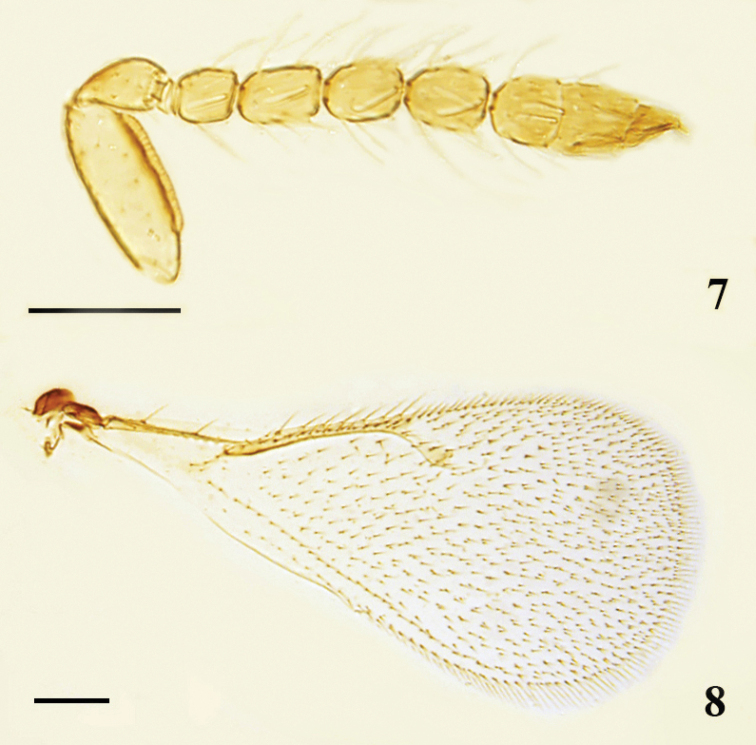
*Oomyzus flavotibialis* sp. nov., paratype male **7** antenna, lateral view **8** forewing, dorsal view. Scale bars: 100 μm.

**Male.** Similar to female. Antenna (Fig. 7) with scape robust, shorter than an eye, 2.62× as long as broad; plaque 0.67× as long as scape; pedicel 1.71× as long as broad; F1 quadrate, shorter than other funicular segments, F2–F4 similar in shape, 1.40× as long as broad; clava broader than funicle, 2.74× as long as broad. Forewing (Fig. 8) with costal cell 1.4× as long as MV, MV 2.5× as long as STV.

##### Host.

Unknown.

##### Distribution.

China (Liaoning, Shandong).

##### Etymology.

*Flavus*, Latin for yellow, golden; in reference to the completely yellow tibiae.

#### Oomyzus gallerucae

Taxon classificationAnimaliaHymenopteraEulophidae

(Fonscolombe)

FDFDA31F-71DF-5DEE-85C0-3174DED02FBB

Figures 9–14

##### Material examined.

China, **Liaoning Province**, Fushun City, 18.VI.2012, Hui Geng, Xiang-Xiang Jin, and Jiang Liu, sweeping [2 females on slides, NEFU].

##### Diagnosis.

**Figures 9–14.**
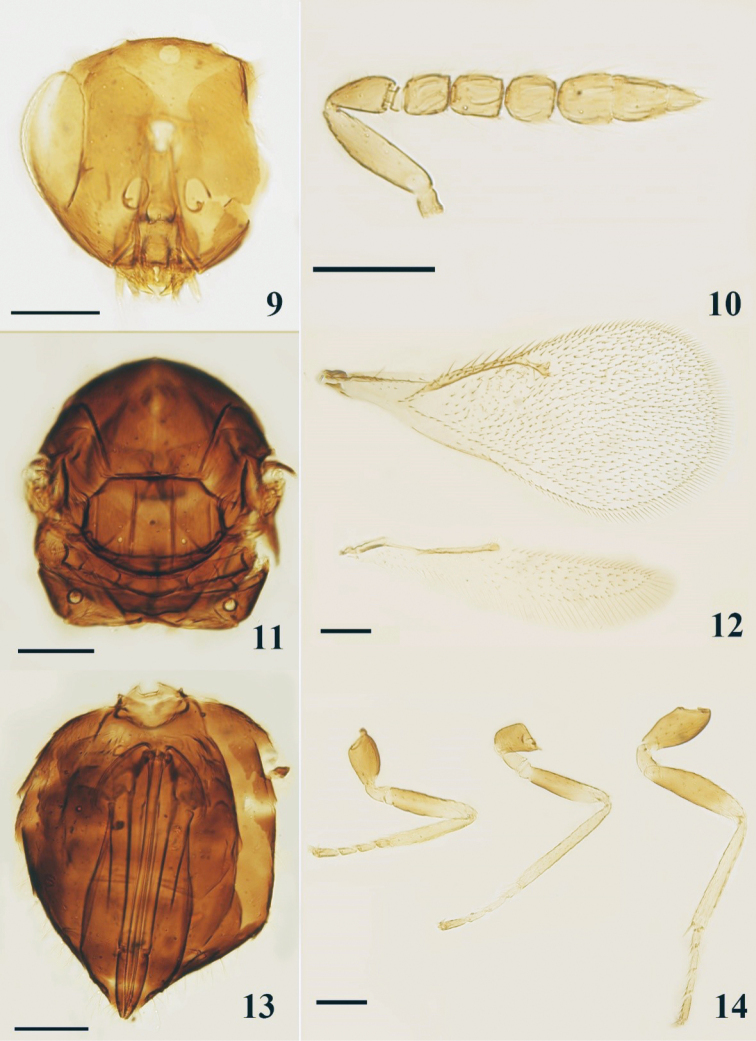
*Oomyzus gallerucae*, female **9** head, frontal view (partly destroyed) **10** antenna, lateral view **11** mesosoma, dorsal view **12** fore- and hind wings, dorsal view **13** metasoma, ventral view **14** legs, lateral view. Scale bars: 100 μm.

**Female.** Head (Fig. 9) with POL about 2× OOL. Antenna (Fig. 10) with funicle segments quadrate or only very slightly longer than wide; clava as long as funicle, 2.7× as long as broad. Mesosoma (Fig. 11) 1.20–1.25× as long as broad. Mesoscutum midlobe with three adnotaular setae on each side in one row, median line present. Propodeum medially as long as or slightly shorter than dorsellum, median carina raised, narrower in front but broadening posteriorly. Forewing (Fig. 12) costal cell as long as or slightly longer than MV; SMV with one dorsal seta; MV 2.8–3.2× STV. **Male.** Unknown for Chinese material.

##### Comments.

Our specimens agree well with the description by Graham (1985). For a redescription and taxonomic history, see Graham (1985).

##### Hosts.

Unknown from China. Outside records include: *Cassida rubiginosa* (Thompson, 1955), *Galerucella lineola* (Herting, 1973), *Galerucella singhara* (Husain & Khan, 1986), *Galerucella xanthomelaena* (Peck, 1963), *Xanthogaleruca luteola* (Meiners & Hilker, 1997) =*Galerucella luteola* (Hesami et al., 2010) = *Pyrrhalta luteola* (Graham, 1985) (Coleoptera: Chrysomelidae)

##### Distribution.

China (Liaoning) [new record], India, Iran, Russia, many countries in Europe, USA, Argentina, Australia.

## Supplementary Material

XML Treatment for Oomyzus flavotibialis

XML Treatment for Oomyzus gallerucae
